# Human Cytomegalovirus UL29/28 Protein Interacts with Components of the NuRD Complex Which Promote Accumulation of Immediate-Early RNA

**DOI:** 10.1371/journal.ppat.1000965

**Published:** 2010-06-24

**Authors:** Scott S. Terhune, Nathaniel J. Moorman, Ileana M. Cristea, John Paul Savaryn, Christian Cuevas-Bennett, Michael P. Rout, Brian T. Chait, Thomas Shenk

**Affiliations:** 1 Department of Molecular Biology, Princeton University, Princeton, New Jersey, United States of America; 2 Department of Microbiology and Molecular Genetics & Biotechnology and Bioengineering Center, Medical College of Wisconsin, Milwaukee, Wisconsin, United States of America; 3 Laboratory for Mass Spectrometry and Gaseous Ion Chemistry, The Rockefeller University, New York, New York, United States of America; 4 Laboratory of Cellular and Structural Biology, The Rockefeller University, New York, New York, United States of America; Oregon Health and Science University, United States of America

## Abstract

Histone deacetylation plays a pivotal role in regulating human cytomegalovirus gene expression. In this report, we have identified candidate HDAC1-interacting proteins in the context of infection by using a method for rapid immunoisolation of an epitope-tagged protein coupled with mass spectrometry. Putative interactors included multiple human cytomegalovirus-coded proteins. In particular, the interaction of pUL38 and pUL29/28 with HDAC1 was confirmed by reciprocal immunoprecipitations. HDAC1 is present in numerous protein complexes, including the HDAC1-containing nucleosome remodeling and deacetylase protein complex, NuRD. pUL38 and pUL29/28 associated with the MTA2 component of NuRD, and shRNA-mediated knockdown of the RBBP4 and CHD4 constituents of NuRD inhibited HCMV immediate-early RNA and viral DNA accumulation; together this argues that multiple components of the NuRD complex are needed for efficient HCMV replication. Consistent with a positive acting role for the NuRD elements during viral replication, the growth of pUL29/28- or pUL38-deficient viruses could not be rescued by treating infected cells with the deacetylase inhibitor, trichostatin A. Transient expression of pUL29/28 enhanced activity of the HCMV major immediate-early promoter in a reporter assay, regardless of pUL38 expression. Importantly, induction of the major immediate-early reporter activity by pUL29/28 required functional NuRD components, consistent with the inhibition of immediate-early RNA accumulation within infected cells after knockdown of RBBP4 and CHD4. We propose that pUL29/28 modifies the NuRD complex to stimulate the accumulation of immediate-early RNAs.

## Author Summary

A key event in regulating gene expression involves changes in the acetylation status of core histones. Regulation is accomplished by a balance between the addition of acetyl groups by histone acetyltransferase enzymes and removal of the moieties by deacetylases. These changes are essential in regulating cellular differentiation and proliferation and, likewise, disruption results in a variety of pathologies, including cancer. In addition, these key regulators are targeted by herpesviruses to ensure persistent infection during the life of the host. In the case of the herpesvirus human cytomegalovirus (HCMV), changes in histone acetylation have been implicated in the choice between latent and acute phases of infection. We have used a focused proteomics approach to identify proteins that are interacting with and regulating the histone deacetylase 1 (HDAC1) protein during acute cytomegalovirus infection. Our studies identified numerous cellular and viral proteins including HCMV pUL29/28. This protein bound to components of the nucleosome remodeling and deacetylase complex, NuRD, and functional NuRD components were necessary for HCMV gene expression and infection. Our study demonstrates a new tool for studying host-pathogen interactions as well as provides new insights into the complex regulation of HDAC1 during HCMV replication.

## Introduction

Human cytomegalovirus (HCMV) is a ubiquitous β-herpesvirus that causes life threatening disease in immunocompromised adults, specifically individuals undergoing solid organ or hematopoietic cell transplant and individuals with Acquired Immunodeficiency Syndrome (AIDS) [1]. In addition, congenital HCMV infections cause life-long disabilities in a significant number of children. In recent years, chronic infection has also been linked to cardiovascular disease (reviewed in [2]) and correlated with a decrease in life expectancy [3]; and the virus has been found in several types of human tumors and it expresses gene products with oncogenic potential (for a review see [4]).

The lytic HCMV replication cycle proceeds through a highly coordinated series of events. At the very start of infection, cellular defenses are inhibited and viral immediate-early gene expression is facilitated by proteins and RNAs that are delivered to cells as constituents of virions [5]–[7]. As soon as the viral genome reaches the nucleus, it expresses immediate-early gene products [8], [9], which also help to establish a permissive environment for replication and activate downstream elements of the viral gene expression cascade [1]. Early genes are expressed next, encoding proteins responsible for viral DNA replication as well as products regulating cellular responses to infection; and, finally, late genes encode for proteins needed to assemble infectious viral particles [1].

Upon entry, the HCMV genome rapidly becomes associated with cellular histones [8], which then undergo dynamic changes in their modification state [9]. During the immediate-early phase of the replication cycle, high levels of histone acetylation are detected by 3 h postinfection (hpi) at immediate-early promoters, including the major immediate-early promoter (MIEP). A slight reduction in MIEP histone acetylation occurs at 12 hpi. The change is mediated by the virus-coded IE2 protein binding to the so-called cis-repressive sequence within the promoter and histone deacetylase 1 (HDAC1) activity [9], [10]. Following the onset of viral DNA replication, a general increase in histone occupancy on the genome occurs [8] with high levels of histone acetylation at early and late promoters, coincident with their enhanced activity [9]. In addition to dynamic histone modifications during the lytic replication cycle, increased histone acetylation at the MIEP correlates with reactivation from latency [11].

HDACs have been indentified within multi-protein complexes. HDAC1 is found in the nucleosome remodeling and deacetylase (NuRD) [12], coREST[13], and Sin3 [14] complexes. NuRD is a large complex containing HDAC1 or 2 and chromatin remodeling ATPases, CHD4 and CHD3 [12]. In addition to these proteins, the core NuRD complex includes RBBP4, RBBP7, MBD3, P66A and P66B, and several members of the metastasis-associated (MTA) protein family. The histone demethylase, LSD1, has also been found within the NuRD complex [15]. The recruitment of the core NuRD complex to specific promoters occurs through interactions with transcription factors [12]. NuRD has been proposed to remodel chromatin through histone deacetylation by HDAC1 and 2, as well as by mediating nucleosome mobility via a CHD3- and CHD4-modulated sliding mechanism [16]. In addition to modifications of histones, the complex deacetylates non-histone proteins, including p53 [17] and HIF-1α [18], which play critical roles in regulating gene expression [12]. Disruption of the NuRD complex has a profound impact on cellular differentiation and proliferation and is associated with metastatic cancer [12].

Regulation of HDACs plays important roles in viral replication and pathogenesis. In HCMV, the virus-coded IE2 protein interacts with HDAC1 [10] and 2 [19] and auto-regulates viral gene expression [10]. Inhibition of HDACs during CMV infection hyperactivates immediate-early gene expression [20]–[24], overcomes the repressive effects of hDaxx at the MIEP [25] and substantially rescues the growth of an IE1-deficient virus [26]. HCMV-coded IE1 protein binds HDAC3 and promotes viral replication by antagonizing histone deacetylation [27]. Proteins from several other herpesviruses bind to and modulate HDACs, including herpes simplex type 1 virus proteins ICP8 [28] and ICP0 [29], [30] and Epstein-Barr virus protein EBNA3C [31], [32]. Beyond herpesviruses, modulation of deacetylases occurs during human papillomavirus (HPV) infection. HPV E7 protein binds [33], [34] and modulates HDAC1, resulting in the activation of E2F-responsive genes [35].

We recently reported that constituents of the NuRD complex can be captured from infected cell extracts with a tagged HCMV UL38-coded protein at 24 hpi [36], identifying a possible interaction between the viral protein and the NuRD complex. To better understand the relationship between histone modification and HCMV gene transcription, we have now performed a global search for putative HDAC1-interacting proteins in the context of infection. We validated several of the interactions, including the formation of complexes between components of the NuRD complex with the HCMV-coded proteins, pUL38 and pUL29/28. pUL38 is an early protein that controls cellular stress responses; it blocks apoptosis [37] and influences both the unfolded protein response [38] and mTOR signaling [36]. pUL29/28 is necessary for efficient viral replication [39], [40] and a pUL29/28-deficient virus fails to express normal levels of immediate-early gene products [41]. Our studies show that the interaction between pUL38 and HDAC requires pUL29/28, and that blocking expression of NuRD components inhibits replication of a wild-type virus. Finally, we demonstrate that full activation of the HCMV MIEP requires pUL29/28 but not pUL38. We propose that HCMV co-opts NuRD function at least in part to enhance expression of its immediate-early genes.

## Materials and Methods

### Cell culture, viral strains and plasmids

All cells were propagated in Dulbecco's modified Eagle medium supplemented with 10% fetal bovine serum and penicillin-streptomycin (Invitrogen) in 5% CO_2_ at 37°C. Primary human foreskin fibroblast (HF; passages 6 to 15) [42] and human MRC-5 embryonic lung fibroblast cells (American Type Culture Collection) were used for HCMV infections, except for the initial identification of HDAC1-interacting proteins, which utilized life-extended human foreskin fibroblasts [43]. Luciferase experiments were completed using the human osteosarcoma cell line, U-2 OS (American Type Culture Collection). A matched pair of pUL38-expressing and control U-2 OS cells were constructed using retrovirus stocks made by transfecting pLXSN (Clontech) or pLXSN-UL38 into Phoenix Ampho cells [44] using FuGENE 6 transfection reagent (Roche). The control U-2 OS cells received an empty retrovirus vector, while U-2 OS pUL38 cells contained a retrovirus expressing HCMV pUL38. Transduced U-2 OS cells were selected for presence of the retrovirus by using Geneticin (Invitrogen). Human embryonic kidney 293T cells (American Type Culture Collection) were used for generating shRNA-expressing lentiviruses as described in RNA interference (RNAi) analysis.

Wild-type HCMV, BAD*wt*, was obtained from an infectious bacterial artificial chromosome clone of the AD169 strain, termed pAD/Cre [45]. The BAD*in*UL29F and BADinUL38Tap viruses as well as the pUL28-, pUL29- and pUL38-deficient mutants, BAD*sub*UL28, BAD*sub*UL29 and BAD*sub*UL38, have been previously described [36], [37], [39], [41]. Viruses were propagated on fibroblasts with the exception of BAD*sub*UL38 which was propagated on pUL38-expressing fibroblasts [37]. Virus preparations were partially purified and concentrated by centrifugation through a sorbitol cushion (20% D-sorbitol, 50 mM Tris-HCl [pH 7.2], 1 mM MgCl_2_). Viral titers were determined by using an immunofluorescence assay to quantify pUL123 (IE1)-expressing cells [37]. Briefly, serial dilutions of virus samples were plated on fibroblasts and cells were fixed and permeabilized in methanol at −20°C for 15 min at 36 hpi. pUL123-positive cells were quantified by using an antibody to pUL123 and a secondary antibody conjugated to Alexa 488 (Invitrogen). For several experiments, cell-free virus was collected from supernatants and infectivity was determined. To determine DNA levels and particle to PFU ratios in virus stocks, quantitative PCR (qPCR) was performed as previously described [37]. The use of the histone deacetylase (HDAC) inhibitor, trichostatin A (TSA) (Cell Signaling Technologies) in HCMV replication studies has been described by Nevels *et al.* [26]. Briefly, fibroblasts were grown to confluency and pretreated for 24 h in the presence or absence of the drug. Treated cells were infected and then cultured in the continuous presence of 300 nM TSA for virus rescue studies or 500 nM TSA for IE1 RNA expression studies [26].

The identification of HDAC1-interacting proteins used fibroblasts (HF-HD1gfp cells) stably expressing an HDAC1-enhanced green fluorescent protein (gfp) fusion protein, in which the gfp was fused at the C-terminal end of the cell protein. These cells were generated by transduction using a retrovirus produced from pLXSN-HDAC1gfp and a pure population of GFP-positive cells was isolated by using a FACSVantage flow cytometer (BD Biosciences). pLXSN-HDAC1gfp was constructed by PCR amplification using Expand High Fidelity PCR System (Roche) of the HDAC1 gene (primers: 5′-ATAGAATTCAAGCTTGCCACCATGGCGCAGACGCAGGGC-3′ and 5′-CCTTGCTCACGGCCAACTTGACCTCCTC-3′) from an expression plasmid kindly provided by E. Seto (University of South Florida). PCR amplification of gfp used an enhanced GFP expression plasmid (Clontech) (primers: 5′-CTCCTCCAGTTCAACCGGGTGAGCAAGGGCGAGGAGCTG-3′ and 5′-TTAAGATCTTACTTGTACAGCTCGTCCA-3′) (Integrated DNA Technologies). The PCR products were combined, amplified using the terminal primers to each gene, and introduced into pLXSN using the restriction enzymes, EcoRI and BamHI. The pUL38 expression vector, pLXSN-UL38, was produced by PCR amplification of the UL38 gene using HCMV genomic DNA (primers: 5′-ATAGAATTCGCCACCATGACTACGACCACGCATAG-3′ and 5′-ATACTCGAGCTAGACCACGACCACCATCTG-3′) and introduced into pLXSN. The myc-tagged pUL29/28 expression vector, pLXSN-UL29/28myc, was constructed by PCR amplification UL29/28 from cDNA produced from RNA isolated at 6 hpi [41] (primers: 5′-ATAGTTAACGCCACCATGGAACAAAAGCTAATATCAGAAGAAGACCTATCCGGCCGTCGCAAG-3′ and 5′-ATAGGATCCTCACGACGCCCCCGTGC-3′). Additional plasmids used in these studies have been described elsewhere, including pCGN-UL29/28 and pCGN-UL29 [41], MIEP-luciferase [41], NFκB-luciferase (Clontech) and ISRE-luciferase (Stratagene).

### Isolation of HDAC1 complexes

HF-HD1gfp cells were grown to ∼70% confluency, infected with BAD*wt* at a multiplicity of 3 PFU/cell, harvested at 24 hpi and frozen in liquid nitrogen as cell pellets as described [36], [46]. Cell lysis was carried out by cryogenic grinding (Retch MM301 Mixer Mill, Retch Inc) at 30 Hz frequency using 10 cycles of 3 min each [47]. The resulting cell powder (0.42 g) was suspended in 5 ml optimized lysis buffer (20 mM K-HEPES pH 7.4, 0.1 M potassium acetate, 2 mM MgCl_2_, 0.1% (v/v) Tween-20, 1 µM ZnCl_2_, 1 µM CaCl_2_, 0.5% Triton-X100, 250 mM NaCl, 1/100 protease inhibitor cocktail (Sigma)), homogenized, and insoluble material was removed by centrifugation. The supernatant was used for affinity purification by incubation with 6 mg magnetic beads pre-conjugated with anti-GFP antibodies for 1 h at 4°C, as described [47]. Isolated proteins were eluted with 700 µl 0.5 N NH_4_OH, 0.5 mM EDTA solution, resolved by 1-D gel electrophoresis on a 4–12% NuPAGE Novex Bis-Tris gel (Invitrogen), and stained with Coomassie Blue (Pierce).

As a control (Figure S1), an equivalent isolation was performed using extracts of HF cells expressing free GFP that were prepared at 24 hpi at a multiplicity of 3 PFU/cell with BAD*wt*.

### Mass spectrometry analyses

**Figure 1:**
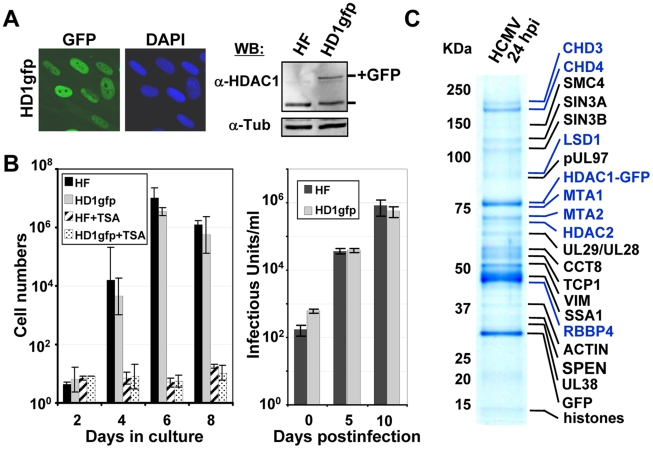
Identification of HDAC1-interacting proteins during HCMV infection. (A) Expression of HDAC1 fused in-frame with the enhanced green fluorescent protein (GFP) in life-extended human fibroblasts. Left panel: The fusion protein was monitored by fluorescence (green), and cells were co-stained with the DNA stain, DAPI (blue). Right panel: Western blot analysis using whole cell lysates from parental (HF) or HDAC1-GFP (HD1gfp) cells and an antibody specific to HDAC1. Anti-tubulin was used as a loading control. (B) HD1gfp cells support efficient HCMV replication. Left panel: Parental (HF) and HD1gfp cell growth were monitored over time and in the presence of 500 nM trichostatin A (TSA), an HDAC inhibitor. Right panel: Replication of wild-type HCMV was quantified by infecting at a multiplicity of 0.01 pfu/cell and determining viral titers from culture supernatants. Data is from duplicate experiments. (C) HDAC1-interacting proteins. HD1gfp cells were infected at a multiplicity of 3 pfu/cell and cell lysates were collected at 24 hpi. HDAC1-GFP interacting proteins were isolated as described in the materials and methods and separated by SDS-PAGE. Gels were stained by Coomassie blue and proteins identified by mass spectrometry. Protein names highlighted in blue represent members of the NuRD complex.

Gel lanes were cut into 25 sections, diced, protein was digested with trypsin (Promega), and the resulting peptides were concentrated on reverse phase resin (POROS 20 R2, Applied Biosystems) and eluted onto a MALDI (matrix assisted laser desorption ionization) target as described [47]. Mass spectrometric analyses were carried out by MALDI MS and MS/MS analyses (single-stage and tandem mass spectrometric analyses, respectively) using a MALDI Qq-TOF (QqTOF Centaur, Sciex) [48] and a MALDI ion trap (LCQDECAXP^PLUS^, Thermo Finnigan) [49], as described [47]. Interacting protein candidates were identified by incorporating the mass spectrometric data in a search against the National Center for Biotechnology Information nonredundant protein database (version 06/10/16) for *Homo sapiens* (152010 sequences) and Viruses (346953 sequences) species using the XProteo (http://www.xproteo.com) algorithm, as described [50]. The limited number of candidate proteins allowed for manual verification of the results, and only those proteins confirmed by MS/MS analyses for at least 2 peptides were considered present (Fig. 1, Table S1). The number of detected peptides, protein sequence coverage, database search score and sequences of confirmed peptides are provided for the isolated protein as supplementary material (Table S1).

### Analysis of protein expression and interactions

Immunoprecipitation experiments were performed as previously described [36]. Briefly, frozen cell pellets were resuspended in 1 ml RIPA buffer (Tris-HCl, 50 mM, pH 7.4; 1% NP-40; 0.25% Na-deoxycholate; 150 mM NaCl; 1 mM EDTA), sonicated and incubated on ice for 30 min. Insoluble material was pelleted by centrifugation and lysates were precleared for 30 min with Protein A/G Sepharose (Santa Cruz) at 4°C. Primary antibody was added for 1 h at 4°C followed by Protein A/G Sepharose for 1 h at 4°C. Following washes, Sepharose-protein complexes were resuspended in sample buffer, boiled at 100°C for 5 min, and samples were resolved on 10% SDS-containing polyacrylamide gels. After transferring proteins to membrane, specific proteins were identified by Western blot performed using indicated antibodies in 1× TBST with 1% BSA as previously described [36]. Secondary antibody conjugated to HRP (Jackson ImmunoResearch) was used at 1∶5000 dilution in 1× TBST plus 1% BSA, and antibody complexes were visualized using ECL Reagent (GE Healthcare).

Chromatin immunoprecipitation (ChIP) analysis was performed as previously described [9]. In brief, at 48 hpi nuclear proteins were cross-linked to DNA with 1% formaldehyde in culture medium for 10 min, after which the reaction was quenched with glycine (0.125 M). Cells were washed and resuspended in buffer containing 10 mM Tris-HCl [pH 8.0], 100 mM NaCl, 1 mM EDTA, 0.5 mM EGTA, 0.1% sodium deoxycholate, 0.5% *N*-lauroylsarcosine, and protease inhibitors [Roche]; and then cells were disrupted and DNA was fragmented by sonication. Aliquots of lysate containing 3 µg of viral DNA were diluted 1∶10 in 0.01% sodium dodecyl sulfate [SDS], 1.1% Triton X-100, 1.2 mM EDTA, 16.7 mM Tris-HCl [pH 8.0], 167 mM NaCl, and protease inhibitors and incubated with antibody overnight at 4°C. The immunocomplexes were captured with protein G-agarose, eluted from the beads at room temperature with buffer containing 1% SDS and 100 mM NaHCO_3_, crosslinks were reversed by incubation at 65°C for 5 h, proteins were digested with proteinase K (40 µg/ml) for 2 h at 55°C, and DNA recovered by using QiaQuick purification columns (Qiagen) and assayed by quantitative PCR (qPCR) with a Power SYBR green PCR kit (Applied Biosystems) and a primer pair specific for the HCMV MIEP (5′-AACAGCGTGGATGGCGTCTCC-3′ and 5′-GGCACCAAAATCAACGGGACTTT-3′). The efficiency of amplification for the primers was determined by generating a standard curve with 10-fold serial dilutions of a known concentration of viral DNA. The slope values of the standard curve for the primer pair amplicons ranged from −3.5 to −3.2, indicating 90 to 100% efficiency. Dissociation curves were performed to ensure that the primers generated a single amplification product, a template control was included, and each sample was run in duplicate.

For immunofluorescent analysis, fibroblasts were grown to confluence on glass coverslips, infected with HCMV at a multiplicity of 0.01 PFU/cell and fixed for 15 min in 2% paraformaldehyde at 24 hpi. Slides were treated for 15 min in 0.1% Triton X-100, washed with PBST (PBS containing 0.2% Tween 20), and incubated for 30 min in PBS-blocking buffer (PBS containing 2% bovine serum albumin and 0.2%Tween 20). Proteins were identified by incubating slides with the indicated primary antibody in PBS blocking buffer for 1 h at room temperature. After further washing with PBST, slides were incubated for 30 min using a secondary conjugated to Alexa 488 or 546 (Invitrogen). Cells were washed with PBST, mounted and viewed using a Zeiss LSM510 confocal microscope (Carl Zeiss). Quantitation of protein colocalization was performed using the Velocity software package (Improvision).

Antibodies used in these studies were: mouse anti-HDAC1 IgG1 (Millipore), rabbit anti-HDAC1 polyclonal (Millipore), rabbit anti-MTA2 polyclonal (Santa Cruz), mouse anti-FLAG M2 IgG1 (Sigma), mouse anti-Myc IgG1(Millipore), rabbit anti-mSin3A polyclonal (Santa Cruz), rabbit anti-TSC2 polyclonal (Santa Cruz), mouse anti-HA IgG1 (Sigma), mouse anti-tubulin IgG1 (Sigma), rabbit anti-protein A (Sigma), mouse anti-IE1 (clone 1B12) [51] mouse anti-pUL38 (clone 8D6) [37] and mouse anti-pUS24 [52].

### Quantitative PCR analysis

RNA levels in infected cells were determined by quantitative real-time reverse transcription-PCR (qRT-PCR) as previously described [37], [41]. Briefly, total RNA was isolated by using TRIzol reagent (Invitrogen), and contaminating DNA was removed from 1.0 µg of total RNA by using DNA-free reagent (Ambion). cDNAs were synthesized from this RNA using Superscript III reverse transcriptase and random hexamers according to the manufacturer's instructions (Invitrogen). DNA was isolated from infected monolayer cultures by harvesting cells and resuspending cell pellets in lysis buffer (400 mM NaCl, 10 mM Tris [pH 8.0], 10 mM EDTA, 0.1 mg/ml proteinase K, 0.2% sodium dodecyl sulfate [SDS]) at 37°C for 12 h. DNA was extracted with phenol-chloroform, extracted again with chloroform, precipitated with ethanol and resuspended in 50 µl water. Real-time PCR analysis was completed using 1 µl of either DNA or cDNA with SYBR green PCR master mix (Applied Biosystems) and primers specific to the sequence of interest. Primers used within these studies have been previously published [41] with the exception of cellular RBBP4 (set 1: 5′-ATAGCCAGTGTGCAGCTCCCTAAT-3′ and 5′-AACGGGCCCTGTTTACTTCTCCTT-3′; set 2: 5′-AGGAAGGCTATGGGCTTTCTTGGA-3′ and 5′-TGCCGTATGCCCTGTAAAGATGGT-3′) and CHD4 (set 1: 5′-AGTGCTGCAACCATCCATACCTCT-3′ and 5′-ATGCCCACCCTCCTTAAGGTTCTT-3′; set 2: 5′-TCGTAAGGGCCTGCGGAATGATAA-3′ and 5′-TTCTCTGATTTGCCTCGCAGGTCT-3′), and firefly luciferase gene (5′-ATTTATCGGAGTTGCAGTTGCGCC-3′ and 5′-GCTGCGAAATGCCCATACTGTTGA-3′). PCR quantitation was performed using the 7900HT Fast Real-Time PCR System (Applied Biosystems). Quantities for unknown samples were defined relative to a standard curve consisting of 10-fold serial dilutions of a single sample and completed for each primer pair. The results were normalized to either β-actin DNA or GAPDH cDNA levels within each sample.

### RNA interference (RNAi) analysis

Disruption of cellular CHD4 and RBBP4 expression utilized short hairpin RNAs (shRNA) stably expressed in primary human fibroblasts or U-2 OS cells. The shRNA clones were expressed from the lentivirus vector, pLKO.1-Puro (Sigma Mission Clones), containing an shRNA sequence against either CHD4 (5′-CCGGGCTGACACAGTTATTATCTATCTCGAGATAGATAATAACTGTGTCAGCTTTTT-3′) or RBBP4 (5′-CCGGCCCTTGTATCATCGCAACAAACTCGAGTTTGTTGCGATGATACAAGGGTTTTTG-3′). The control shRNA consisted of a scrambled sequence (5′-TCAGTCGCGTTTGCGACTGGTTCAAGAGACCAGTCGCAAACGCGACTGTTTTTGGAAAC-3′) and was a kind gift from P. Traktman (Medical College of Wisconsin). Recombinant lentiviruses were produced by co-transfecting 293T cells with lentivirus expression plasmid and packaging plasmids [53], [54] using FuGENE 6 transfection reagent. Infectious lentiviruses were harvested at 48 and 72 hpi, cleared of 293T cells and frozen at −80°C. The infection of fibroblasts and U-2 OS cells with shRNA lentivirus was carried out by adding lentivirus to the cell culture medium and incubating with target cells for 24 h. shRNA-expressing cells were selected by using puromycin at 1 µg/ml (Invitrogen). The inhibition of CHD4 and RBBP4 expression was quantified by qRT-PCR and two separate sets of primers to each gene as listed above.

### Luciferase assays

Control or pUL38-expressing U-2 OS cells were seeded onto 12-well plates and transfected using FuGENE 6 according to the manufacturer's instructions. Cells were transfected with 50 ng of pGL3-MIEP reporter plasmid and 10, 100, or 500 ng of pCGN empty vector or pCGN-pUL29/28 effector plasmid. For ISRE and NF-κB luciferase reporter plasmids, 100 ng of reporter was transfected into pUL38-expressing U-2 OS cells with either 500 ng of control or pUL29/28 effector plasmids. Luciferase activity was assayed 48 h posttransfection using a luciferase reporter assay system (Promega) and a Victor3 luminometer (Perkin-Elmer). Activity was measured by using equal protein amounts within each lysate and normalized to the luciferase activity from empty vector. Expression of pUL29/28 and pUL38 was assayed by Western blot analysis using the same lysates.

## Results

### Identification of HDAC-1 interacting proteins during HCMV infection

As discussed above, regulation of the viral chromatin state, including deacetylation of histones, plays a pivotal role in efficient HCMV infection. We were interested in determining what viral proteins might influence these events through interactions with HDACs. We focused on HDAC1, a 55 kDa nuclear protein. HDAC1 previously has been shown to influence herpesvirus replication as well as latency [30], [32], [55]–[57].

We constructed a retrovirus vector expressing the neomycin resistance gene and the HDAC1 open reading frame (ORF) with the enhanced green fluorescent protein (GFP) ORF fused at its C-terminus (pHDAC1gfp). For these studies, we used life-extended fibroblasts, which can be maintained for extended periods in culture and were demonstrated to support efficient HCMV replication [43]. Cells were transduced using the HDAC1-GFP retrovirus, cells containing the retrovirus were selected by treatment with G418, and a population expressing a moderate level of the fusion protein was isolated by FACS. We observed fluorescence within the nuclei of >99% of the pHDAC1gfp-expressing cells, which are termed HF-HD1GFP cells (Fig. 1A, left panels). Western blot analysis, performed on whole cell lysates from life-extended fibroblasts or HF-HD1GFP cells by using an antibody to HDAC1, demonstrated expression of a 55 kDa endogenous HDAC1 in both cell populations and an additional 75 kDa HDAC1-GFP exclusive to the transduced cells (Fig. 1A, right panel). The 75 kDa protein, pHDAC1gfp, was present at about half the level of endogenous protein, providing confidence that abnormal interactions due to overexpression of the fusion protein are not likely to occur.

Overexpression of HDAC proteins occurs in many cancers, and can affect cell proliferation [58]. To test whether constitutive pHDAC1-gfp expression altered the growth phenotype of the fibroblasts, we monitored their growth rate and found that the proliferation of HF-HD1GFP cells was indistinguishable from the parental HF cells (Fig. 1B, left panel). In addition, HF-HD1GFP cell growth was inhibited by trichostatin A (TSA), a class I and II HDAC inhibitor (Fig. 1B, left panel). To determine whether expression of HDAC1-GFP influenced HCMV replication, we completed a multistep growth analysis (Fig. 1B, right panel). Parental HF or HF-HD1GFP cells were infected at a multiplicity of 0.01 pfu/ml and medium was collected after various time intervals. A similar amount of infectious virus was released from the two cell lines indicating that expression of pHDAC1gfp does not affect HCMV replication. We conclude that HF-HD1GFP cells are a suitable cell line to identify HDAC1-interacting proteins during HCMV infection.

To identify cellular and viral proteins that interact with HDAC1, we infected HF-HD1GFP cells with wild-type HCMV (BAD*wt*). At 24 hpi, pHDAC1gfp and associated proteins were isolated from cell extracts by using one-step immunoaffinity purification on magnetic beads coated with antibodies to GFP [36], [46], [47]. Isolated viral and host proteins were resolved by electrophoresis and identified by mass spectrometry. The major protein bands evident in the Coomassie blue-stained polyacrylamide gel were identified by employing sequential MALDI QqTOF MS and MALDI IT MS/MS analyses (Fig. 1C), and a complete listing of these proteins is presented in Table S1. Although their capture with pHDAC1gfp suggests they associate with HDAC1, confirmation is needed to definitively identify the proteins as interacting partners. Therefore, the proteins in table S1 must be considered as *potential* HDAC1-interacting proteins.

The HCMV proteins pUL29/28, pUL38, and pUL97 were identified in the capture experiment (Fig. 1C and Table S1). pUL97 is the 78 kDa viral kinase that is necessary for efficient replication and viral egress [59], [60]. pUL29/28 is a 79 kDa nuclear protein expressed from a spliced RNA containing the UL28 and UL29 ORFs, which is packaged in viral particles [61]. Disruption of pUL29/28 results in a virus that replicates poorly and expresses reduced levels of immediate-early genes [39], [40], [61]. pUL38 blocks cells from dying by apoptosis during infection [37]. It functions, in part, by regulating the unfolded protein response [38] and mTOR signaling pathways [36].

Our analysis also identified numerous cellular proteins predicted to interact with HDAC1 (Table S1). Analysis of infected fibroblasts expressing gfp alone (Fig. S1) and studies using different viral proteins tagged with gfp [62] did not detect these proteins, suggesting that they are unique to HDAC1gfp and not the gfp sequence itself. HDAC1 is found in several multi-protein complexes including NuRD [12], coREST [13], and Sin3 [14] complexes. Several proteins previously identified in these complexes were found in our capture experiment. We observed associations with components of the NuRD complex, including HDAC1, HDAC2, MTA1, MTA2, CHD3, CHD4, RBBP4 and LSD1. HDAC1, HDAC2, and RBBP4 are also elements of the Sin3 complex, and additional Sin3 components observed in our studies included SIN3A, SIN3B, and SDS3. HDAC1, HDAC2, and LSD1 are also elements of the coREST complex. In addition, putative interactions were observed with several cellular proteins previously not known to interact with HDAC1 (Table S1). Finally, we observed free GFP among the captured proteins, and this was not surprising since our past studies have demonstrated that loss of a portion of the epitope tag from fusion proteins occurs during HCMV infection [36], [62].

In a previous study we identified, but did not confirm, predicted associations of pUL38 with pUL29/28, HDAC1 and several additional components of the NuRD complex [36], and we have demonstrated that both pUL29/28 and pUL38 are required for efficient HCMV replication [37], [61]. With these earlier results in mind, we focused on the predicted association of HDAC1 with the viral proteins pUL29/28 and pUL38.

### HCMV pUL29/28 and pUL38 interact with components of the NuRD complex

**Figure 2:**
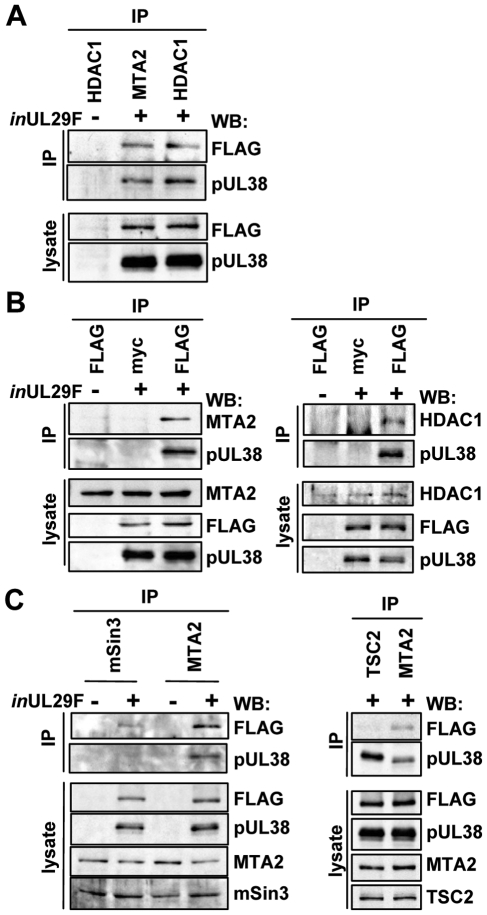
HCMV pUL29/28 and pUL38 specifically bind to constituents of the NuRD complex. Fibroblasts were infected at a multiplicity of 3 pfu/cell using BAD*in*UL29F (*in*UL29F) virus, and whole cell lysates were prepared 24 h later. (A) HDAC1 and MTA2 coprecipitate with pUL29/28 and pUL38. Immunoprecipitations (IP) were performed using antibodies to MTA2 or endogenous HDAC1. Western blot (WB) analysis employed an anti-FLAG (pUL29/28) or pUL38 antibodies, and included analysis of lysates as a control. An uninfected control sample was also included. (B) pUL29/28 coprecipitates HDAC1, MTA2 and pUL38. Left panel: The immunoprecipitation was carried out using an anti-FLAG antibody (pUL29/28) or an isotype-specific anti-myc control antibody followed by Western blot analysis using antibodies to pUL38 and MTA2. Right panel: The experiment was repeated using antibody to HDAC1. (C) pUL29/28 interacts with the NuRD and Sin3 complexes. Left panel: Immunoprecipitation from whole cell lysates from uninfected or BAD*in*UL29F (*in*UL29F) infected cells using antibodies to mSin3A and MTA2. Western blot analysis with antibodies against FLAG (pUL29/28), pUL38, MTA2 or mSin3A. Right panel: No evidence for interaction of pUL29/28 with tuberous sclerosis protein 2 (TSC2). Immunoprecipitations used antibodies against TSC2 and MTA2 and Western blots were performed with antibodies to FLAG (pUL29/28).

To validate the predicted interactions of pUL29/28 and pUL38 with HDAC1, we performed immunoprecipitation experiments from lysates of normal primary fibroblasts that did not express the HDAC1gfp fusion protein using an antibody to endogenous HDAC1. Because no antibody exists to HCMV pUL29/28, we infected fibroblasts with BAD*in*UL29F, which expresses pUL29/28 with an amino-terminal FLAG epitope and replicates like wild-type virus [61]. Upon immunoprecipitation of endogenous HDAC1, we observed associations with both pUL29/28 and pUL38 at 24 hpi by Western blot assay using anti-FLAG and anti-pUL38 antibodies, respectively (Fig. 2A). Neither protein was detected in lysates from uninfected cells.

To test whether pUL29/28 and pUL38 associate with additional components of the NuRD complex, we repeated the analysis using an antibody to the MTA2 protein, a specific component of the complex [12]. Western blot analysis demonstrated that both pUL29/28 and pUL38 interact with MTA2 (Fig. 2A). We performed the reciprocal immunoprecipitation experiment to confirm the interactions (Fig. 2B, left panel). Fibroblasts were infected with BAD*in*UL29F, pUL29/28 was immunoprecipitated through its FLAG epitope, and Western blot assays detected MTA2 and pUL38 in the precipitates. We also confirmed the interaction between pUL29/28 and HDAC1 using this approach (Fig. 2B, right panel). These interactions were not detected using an isotype-specific control antibody to the myc epitope, arguing for specificity of the immunoprecipitation. We conclude that HDAC1 and a second component of the NuRD complex, MTA2, interact either directly or indirectly with both HCMV pUL29/28 and pUL38 proteins during infection.

Our isolation of HDAC1-interacting proteins detected multiple components of both the NuRD and Sin3 complexes (Fig. 1 and Table S1). We next asked if pUL29/28 and pUL38 associate specifically with components of the NuRD complex or both HDAC1-containing complexes. Antibodies to SIN3A, a constituent of Sin3, or MTA2, a subunit of NuRD, were used to test for coprecipitation of the viral proteins. Both pUL29/28 and pUL38 precipitated with MTA2, while a low level of pUL29/28 and no detectable pUL38 were observed interacting with mSin3 (Fig. 2C, left panel). Thus, pUL29/28 interacts with HDAC1 in association with elements of at least two complexes, NuRD and Sin3. Its interaction with the NuRD constituent appears to be more robust than with the Sin3 element, although this could result in part from differences in performance of the two antibodies used for immunoprecipitation or from reduced accessibility of mSin3 to the antibody when pUL29/28 is present in the complex.

Next, we tested whether pUL29/28 binds to the TSC2 subunit of the tuberous sclerosis protein complex, because we previously demonstrated that pUL38 binds to TSC2 [36]. A TSC2-specific antibody coprecipitated pUL38 from infected cells, but very little pUL29/28, whereas the anti-MTA2 antibody clearly coprecipitated both pUL38 and pUL29/28 (Fig. 2C, right panel). Thus, the two viral proteins do not always track together, but HDAC1 clearly interacts with both pUL29/28 and pUL38 in the HDAC1-NuRD complex.

**Figure 3:**
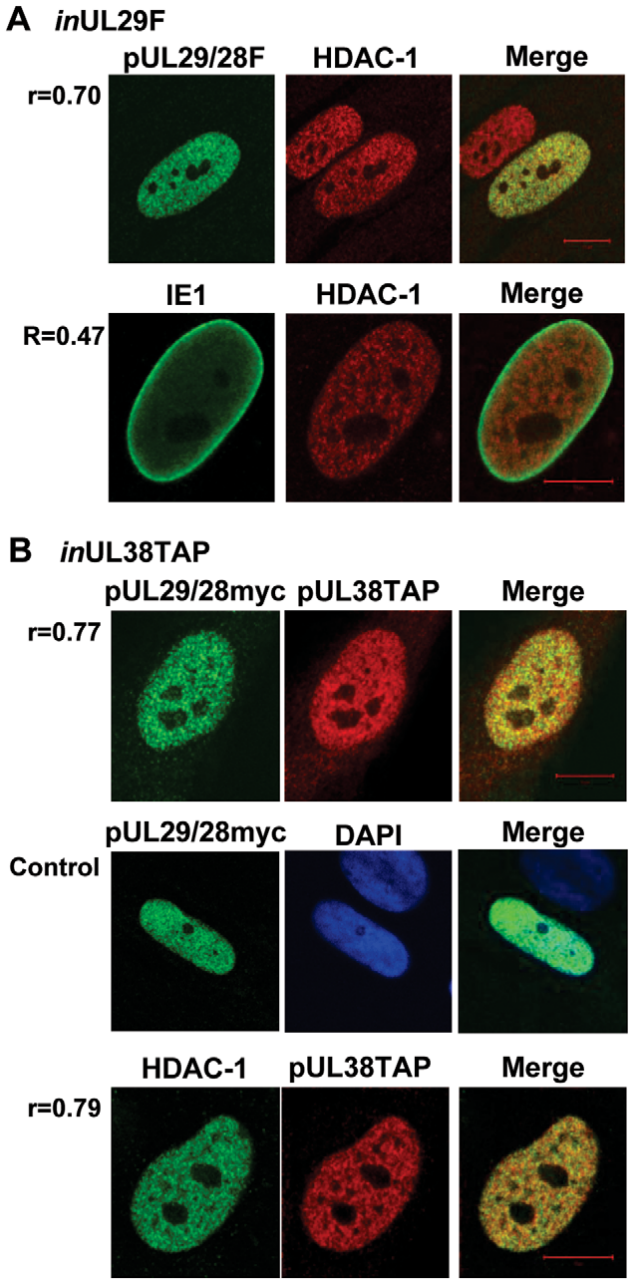
Colocalization of pUL29/28 with cellular HDAC1 and HCMV pUL38. Colocalization is demonstrated within the merged images and quantified using Pearson's correlation (r). (A) BAD*in*UL29F-infected cells (*in*UL29F). Cells were infected at a multiplicity of 0.5 infectious unit/cell, fixed at 24 hpi, and processed for immunofluorescence. Top row shows colocalization of pUL29/28 using an anti-FLAG antibody (green) and of HDAC1 (red). Bottom row demonstrates localization of IE1 (green) and HDAC1 (red) at 24 hpi. (B) BAD*in*UL38TAP-infected cells (*in*UL38TAP). Cells were infected using the pUL38TAP expressing virus and fixed at 24 hpi. Top row shows expression of myc-tagged pUL29/28 using an antibody to the myc epitope (green) and of pUL38 using an antibody to protein A, detecting the TAP sequence (red). Middle row demonstrates the specific detection of pUL29/28myc (green) in the presence of uninfected cells marked by DAPI (blue). Bottom row shows colocalization of HDAC1 (green) and pUL38 (red). Pearson's correlation (r) for colocalization of fluorescent signals was determined for indicated images.

To further investigate the interaction of HDAC1 with pUL29/28 and pUL38, we performed an immunofluorescence microscopy analysis. Visual inspection of the resulting images using cells infected with the BAD*in*UL29F virus showed that pUL29/28 and HDAC1 exhibit significant colocalization within the nucleus at 24 hpi (Fig. 3A, upper panels). No pUL29/28 signal was observed in an uninfected cells (data not shown) as well as an uninfected cell within the same image (Fig. 3A, upper panels), demonstrating specificity of the FLAG antibody to tagged pUL29/28. Quantitative measurement of images confirmed the colocalization [36], [63], demonstrating that Pearson's correlation (r) was greater for pUL29/28 and HDAC1 (r = 0.70) than for another HCMV nuclear protein, IE1, and HDAC1 (r = 0.47), which was concentrated near the edges of infected nuclei as described previously [52], [64]. Perfect colocalization of two proteins yields r = 1.0 [36]. We also observed colocalization between pUL29/28 and pUL38 within the nucleus (r = 0.77) (Fig. 3B, upper panels). This assay used the BAD*in*UL38TAP virus expressing epitope tagged pUL38 [36] and primary fibroblasts expressing a myc-tagged pUL29/28. No signal for myc-tagged pUL29/28 was observed in a neighboring cell from an uninfected control sample (Fig. 3B, middle panels), demonstrating specificity of the antibody to pUL29/28. Finally, pUL38 and HDAC1 were colocalized (r = 0.79) at 24 hpi (Fig. 3B, lower panels). These correlation values are consistent with values observed for known protein-protein interactions [36]. Taken together, our results demonstrate that HDAC1 specifically interacts with both pUL29/28 and pUL38. This interaction likely results from an association of the viral proteins with the NuRD and Sin3 complexes.

### pUL29/28 is necessary for pUL38 binding to HDAC1

**Figure 4:**
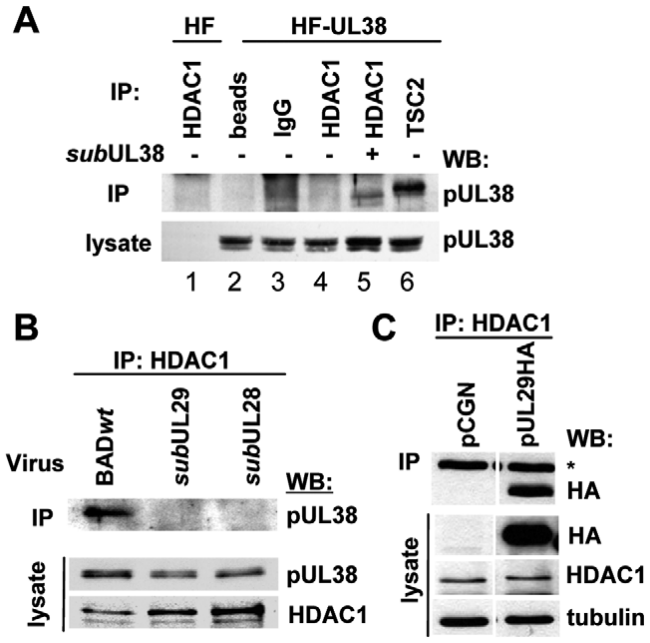
HCMV pUL29/28 is necessary for the HDAC1 interaction with pUL38. (A) The interaction between pUL38 and HDAC1 requires additional factors present during viral infection. Immunoprecipitation (IP) from whole cell lysates isolated from either uninfected control cells (HF) or pUL38-expressing cells (HF-UL38) using antibodies against HDAC1 (lanes 1, 4) or TSC2 (lane 6). Immunoprecipitation of HDAC1 (lane 5) was also completed upon infection of HF-UL38 cells with a pUL38-deficient virus, BAD*sub*UL38 (*sub*UL38) at 24 hpi. Binding to pUL38 was detected by Western blot (WB) analysis with an anti-pUL38 antibody. Beads without antibody and normal IgG were used as specificity controls (lane 2, 3). (B) pUL38 binding to HDAC1 requires pUL29/28. Cells were infected at 3 pfu/cell using wild-type (BAD*wt*), BAD*sub*UL29 (*sub*UL29) or BAD*sub*UL28 (*sub*UL28) mutant viruses and whole cell lysates collected at 24 hpi. Interactions were detected by immunoprecipitation of HDAC1 and Western blot to pUL38. Lysate controls demonstrate expression of pUL38 in all samples. (C) pUL29 binds HDAC1 in the absence of infection. Expression vectors pCGN or pCGN-pUL29HA (pUL29HA) were transfected into U-2 OS cells and whole cell lysates were collected 48 h later. Binding was demonstrated by immunoprecipitation of HDAC1 and Western blot to HA. Lysate controls show expression of pUL29HA, HDAC1 and tubulin, and an asterisks indicates heavy chain of IgG.

pUL29/28 plus pUL38 were coprecipitated with antibodies against HDAC1, and pUL38 plus HDAC1 were precipitated with pUL29/28 antibody, arguing that these proteins exist within the same complex. We next tested whether pUL38 depends on pUL29/28 for interaction with HDAC1 by performing coimmunoprecipitations using uninfected fibroblasts expressing pUL38. Using a pUL38-specific antibody to assay by Western blot, we observed the viral protein in lysates of pUL38-expressing cells (Fig. 4A, lane 2–6). However, we did not detect an interaction between pUL38 and HDAC1 (Fig. 4A, lane 4), although in a control experiment, pUL38 was co-precipitated by using TSC2-specific antibody (Fig. 4A, lane 6). To determine the role of additional viral proteins in mediating this interaction, we infected pUL38-expressing cells with a pUL38-deficient virus [37]. We repeated the immunoprecipitation of HDAC1 and in this case we detected pUL38 by Western blot assay (Fig. 4A, lane 5). pUL38 is expressed in several isoforms of varying molecular weight due to the use of alternative starting methionines [36], and it appears that lower molecular weight forms preferentially interact with HDAC1 whereas higher molecular weight forms interact mainly with TSC2 (Fig. 4A, compare lanes 5 and 6). This data indicates that another viral protein or cellular protein induced during infection mediates the interaction between pUL38 and HDAC1.

Because pUL29/28 interacts with both pUL38 and HDAC1 (Fig. 2), we next asked whether pUL29/28 is necessary for the interaction between pUL38-HDAC1. We infected fibroblasts at a multiplicity of 3 pfu/cell with BAD*wt* and two mutant viruses containing disruptions in either the UL28 (BAD*sub*UL28) or UL29 (BAD*sub*UL29) ORFs, both of which fail to express normal levels of immediate-early genes [39], [61]. pUL38 was coprecipitated with an HDAC1-specific antibody from whole cell lysates after infection with wild-type virus but not pUL29/28-deficient viruses (Fig. 4B). From these results, we conclude that pUL29/28 is necessary for the interaction between pUL38 and HDAC1 during infection. Next, we asked whether any additional viral proteins are needed for the interaction between HDAC1 and pUL29/28. For these studies, we used the smaller isoform of the protein since we were unable to isolate the full length protein from uninfected cells under conditions that were compatible with immunoprecipitation experiments. We transiently expressed pUL29 containing a hemagglutinin epitope tag in U-2 OS cells [41]. Under these conditions, HCMV pUL29 was co-immunoprecipitated with HDAC1 (Fig. 4C), while no band at the appropriate molecular weight was observed using the empty vector control (Fig. 4C). We conclude that no additional viral proteins are necessary to mediate the interaction of pUL29/28 and HDAC1, and that the critical region within the viral protein needed for the interaction is present within the UL29-coded domain.

**Figure 5:**
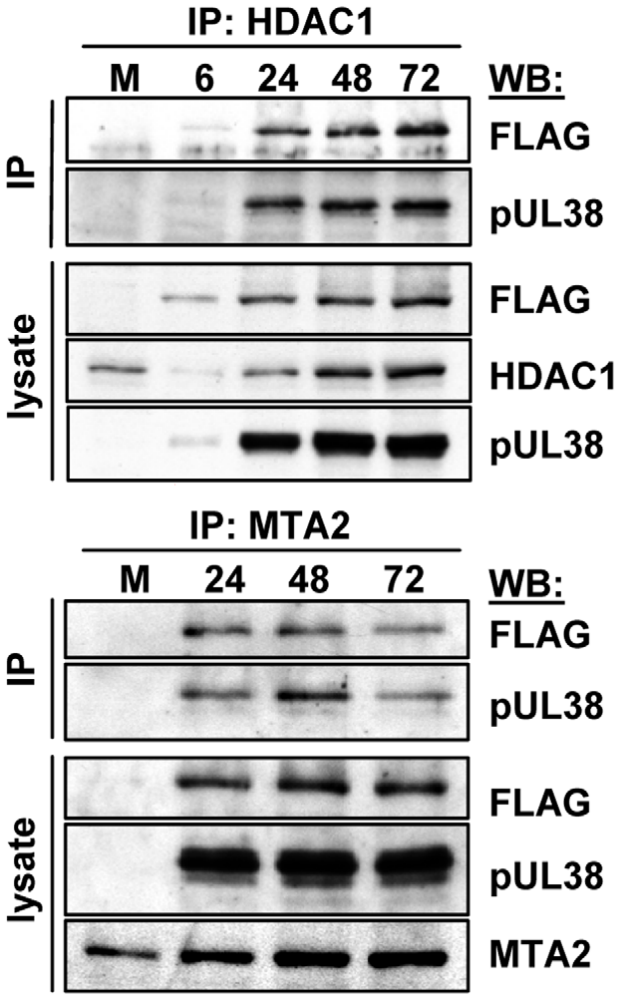
Accumulation of protein complexes during infection with BAD*in*UL29F. Fibroblasts were infected at a multiplicity of 3.0 pfu/cell and harvested at the indicated times. NuRD complexes were isolated by immunoprecipitation (IP) using an anti-HDAC1 antibody (top panels) or an anti-MTA2 antibody (bottom panels) and interacting proteins determined by Western blot (WB) assay using antibodies to pUL38, HDAC1, MTA2 and the FLAG epitope in pUL29/28.

pUL38 is exclusively nuclear at 8 hpi, but present in both the nucleus and cytoplasm at 24 hpi [36], [37]; in contrast pUL29/28 is expressed exclusively within the nucleus through 48 hpi and then in nucleus and cytoplasm at 72 hpi [61]. To determine whether the pUL29/28-pUL38-HDAC1 complex is formed during a defined time over the course of HCMV infection, i.e., when both viral constituents are clearly in the nucleus, we infected fibroblasts with BAD*in*UL29F and collected whole cell lysates during the course of HCMV infection beginning at 6 hpi. Using an antibody against HDAC1, both pUL29/28 and pUL38 were co-precipitated beginning at 6 hpi and at all times tested through 72 hpi (Fig. 5, upper panel). We confirmed the presence of the complex at late times by using antibody to the MTA2 constituent of the NuRD complex (Fig. 5, lower panel). The pUL29/28-pUL38-HDAC1 complex forms very early during infection and exists through late times, presumably reflecting the fact that both viral proteins are present in the nucleus at all times after infection that were tested.

### pUL29/28 and pUL38-deficient viruses are not rescued by inhibition of deacetylase activity

**Figure 6:**
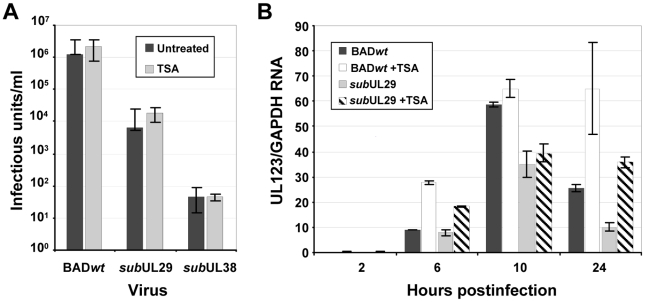
Inhibition of deacetylase activity fails to rescue the replication of pUL29/28 or pUL38-deficient viruses. (A) Trichostatin A does not rescue pUL29/28 or pUL38-deficient viruses. Fibroblasts were infected at a multiplicity of 0.1 pfu/cell using wild-type (BAD*wt*) or inoculum containing equivalent numbers of viral genomes for BAD*sub*UL29 (*sub*UL29) or BAD*sub*UL38 (*sub*UL38) viruses. Cultures were treated with 300 nM TSA for the duration of the experiment and viral titers from culture supernatants were determined at 10 dpi. Data is from duplicate experiments. (B) BAD*sub*UL29 (*sub*UL29) virus is deficient in immediate-early gene expression irrespective of treatment with TSA. Cells pretreated with 500 nM of TSA were infected using 0.1 pfu/cell of wild-type (BAD*w*t) or BAD*sub*UL29 (*sub*UL29) virus as described above, and total RNA was harvested at the indicated times. Expression of IE1 RNA was determined by qRT-PCR using cDNA produced from DNase-treated RNA and random hexamers. Quantitation was completed with SYBR green and primers specific to exon 4 of IE1. Samples were normalized to cellular GAPDH RNA levels.

It is well established that inhibition of HDACs during cytomegalovirus infection stimulates immediate-early gene expression [20]–[24], [65]. Consequently, we reasoned that, if pUL29/28 or pUL38 interacts with HDAC1 to inhibit its activity, then an HDAC inhibitor might compensate for loss of pUL29/28 or pUL38 function. To test this possibility, fibroblasts were infected with BAD*wt*, a pUL38-deficient (BAD*sub*UL38) or pUL29/28-deficient (BAD*sub*UL29) virus in the presence or absence of the HDAC antagonist, trichostatin A (TSA). Because we previously observed an abnormality in particle to PFU ratio for BAD*sub*UL38 [37], we infected fibroblasts at a multiplicity of 0.1 pfu/cell for the wild-type virus and used an equivalent number of particles for the mutants. Culture medium was collected at 10 days post infection and viral titers were determined by quantifying the number of IE1-positive fibroblasts generated by a unit volume of virus (Fig. 6A). In the absence of drug, we observed an ∼10^2^-fold decrease in virus yield for BAD*sub*UL29 and an ∼10^4^-fold decrease for BAD*sub*UL38, consistent with our previous observations [37], [61], and the addition of TSA did not significantly rescue the growth of either mutant.

The absence of pUL29/28 results in reduced levels of immediate-early gene expression at very early times during infection [61]. To determine if inhibiting HDAC activity could rescue this phenotype, we infected cells pretreated with 500 nM of TSA [26], [66] with BAD*wt* or BAD*sub*UL29 and quantified IE1 RNA accumulation (Fig. 6B). The pUL29/28-deficient mutant generated reduced levels of RNA as compared to wild-type virus in the absence of TSA as previously seen [61]. Upon addition of TSA, a similar increase in IE1 levels was observed in both wild-type and BAD*sub*UL29 infected cells and, importantly, TSA did not restore expression of the immediate-early RNA within mutant virus-infected cells to wild-type levels. Chemical inhibition of HDAC activity does not rescue the growth defect that occurs during replication in the absence of pUL29/28 or pUL38.

### Multiple constituents of the NuRD complex are needed for efficient HCMV replication

**Figure 7:**
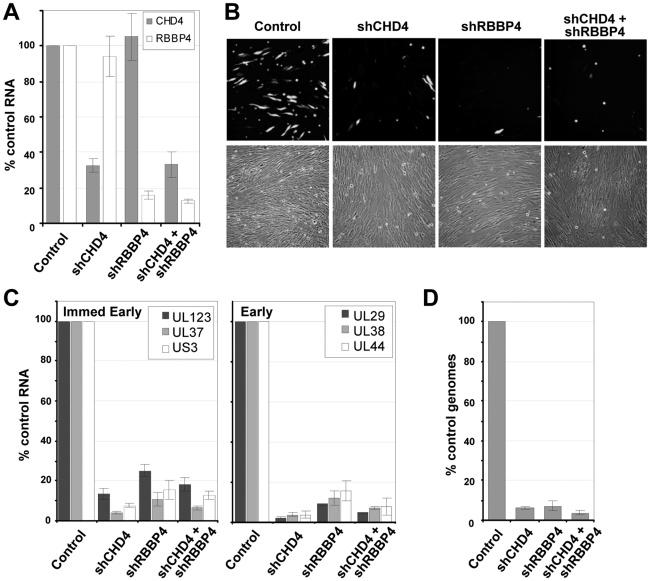
Reduced expression of components of the NuRD complex inhibits HCMV replication. (A) Disruption of NuRD complex in primary fibroblasts. Short hairpin RNA (shRNA) sequences to a scrambled control, CHD4, RBBP4 or both CHD4 and RBBP4 were delivered to fibroblasts using lentivirus vectors and expressing cells were isolated by puromycin resistance. Expression of CHD4 and RBBP4 was quantified by qRT-PCR using two separate sets of gene-specific primers and total cellular RNA. The data was normalized to GAPDH RNA levels and includes the average percent reduction for both primer sets to each gene. (B) shRNA-expressing cells were infected at a multiplicity of 0.25 pfu/cell with a BAD*wt* derivative expressing GFP. Images of infected cells were captured at 96 hpi. (C) Expression of immediate-early and early RNAs at 10 hpi was determined by qRT-PCR using cDNA produced from DNase-treated RNA and random hexamers. Quantitation was completed with SYBR green and sequence-specific primers as indicated. Expression was normalized to cellular GAPDH RNA levels. (D) HCMV genome replication was quantified by qPCR at 96 hpi. qPCR data was normalized using primers to β-actin. Data is derived from replicate experiments.

Since inhibition of HDAC activity did not rescue the mutant viruses, we tested the possibility that NuRD components are needed by HCMV. The NuRD complex was disrupted by using short hairpin RNAs (shRNAs) targeting two specific components of the complex that were detected in the pHDAC1gfp capture experiment: CHD4 and RBBP4 (Fig. 1C). This approach has been successfully used by others to evaluate NuRD function [67], [68]. shRNAs specific for CHD4 or RBBP4 along with a non-specific scrambled sequence were introduced into primary human fibroblasts using a lentivirus vector containing the puromycin-resistance gene, and shRNA-expressing cells were selected by treating cultures with the drug. To demonstrate gene silencing, we assayed the steady state levels of CHD4 and RBBP4 RNA by quantitative RT-PCR (qRT-PCR) using two separate primer sets for each transcript. shRNA specific for CHD4 resulted in a ∼70% reduction in RNA levels as compared to the scrambled control, shRNA against RBBP4 resulted in an ∼85% reduction, and expression of the two shRNAs together generated a similar reduction (Fig. 7A).

To test for a role of the NuRD subunits in HCMV replication, we infected fibroblasts expressing the scrambled control, CHD4, RBBP4 or both shRNAs with wild-type virus expressing a GFP marker gene at a multiplicity of 0.25 PFU/cell, and visualized GFP-positive cells 96 h later (Fig. 7B). The shRNAs against CHD4, RBBP4, or both components of the NuRD complex dramatically reduced the production of GFP-positive cells as compared to the control shRNA. To evaluate the impact on HCMV replication, we quantified both viral gene expression and genome replication during infection. Using qRT-PCR, we observed an ∼80% reduction in the accumulation of immediate-early RNAs (UL123, UL37 exon 1 and US3) and a >90% reduction in early RNAs (UL29, UL38 and UL44) at 10 hpi upon disruption of the NuRD complex as compared to control cells (Fig. 7C). These changes were accompanied by a >90% decrease in HCMV DNA accumulation at 96 hpi (Fig. 7D). We conclude that CHD4 and RBBP4, and by extension very likely a functional NuRD complex, are required for efficient HCMV replication.

### pUL29/28 activates the major immediate-early promoter (MIEP) independently of pUL38

**Figure 8:**
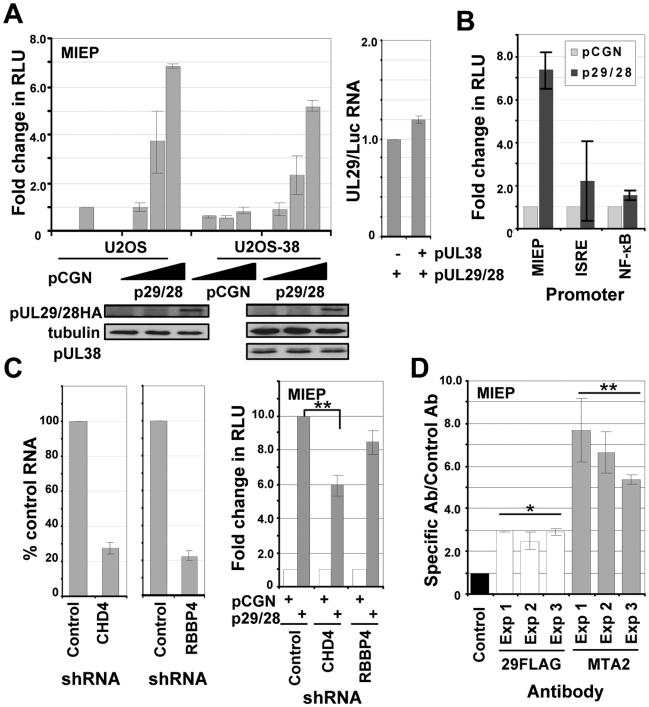
pUL29/28 specifically activates the MIE promoter irrespective of HCMV pUL38 expression. (A) pUL29/28 activates the MIEP. Upper left panel: U2OS cells that stably maintained the empty pLXSN vector (U2OS) or pLXSN expressing HCMV pUL38 (USOS-38) were transfected with 50 ng of pGL3-MIEP reporter plasmid and 10, 100, or 500 ng of pCGN empty vector or pCGN-pUL29/28 effector plasmid. Luciferase activity was assayed 48 h posttransfection using equal protein amounts within each lysate and normalized to luciferase activity from empty vector. Lower panels: The levels of pUL29/28HA and pUL38 expression were assayed by Western blot analysis using the same lysates and antibody to HA, pUL38 or tubulin. Right panel: UL29 and luciferase RNA expression was determined by qRT-PCR from U2OS as compared to U2OS-38 cells. (B) pUL29/28 exhibits promoter-specific effects. Luciferase assays were completed using 500 ng of pCGN or pCGN-pUL29/28 and MIEP, ISRE and NF-κB promoter reporter constructs. The relative luciferase activity was determined as described above. (C) NuRD is required for optimal expression of an MIEP reporter. Left panel: Disruption of the NuRD complex in U-2 OS cells. Short hairpin RNA (shRNA) sequences to a scrambled control, CHD4 or RBBP4 were delivered to U-2 OS cells using lentiviruses and expressing cells were isolated by puromycin resistance. Expression of CHD4 and RBBP4 was quantified by qRT-PCR. The data was normalized to GAPDH RNA levels and includes the percent reduction for each gene relative to control. Right panel: Luciferase assays were completed using either empty pCGN or pCGN-pUL29/28 and MIEP reporter. The relative luciferase activity was determined as described above and the data is derived from replicate experiments (**p<0.03). (D) pUL29/28 and MTA2 interact with the MIE promoter during infection. Cells were infected using BAD*in*UL29F at a multiplicity of 3 pfu/cell and harvested at 48 hpi. Antibodies to the FLAG epitope in pUL29/28FLAG, cellular MTA2 and a control antibody to HCMV pUS24 were used to immunoprecipitate chromatin-DNA complexes, and isolated DNA was quantified by qPCR and primers to the HCMV MIE promoter. The results are plotted as the ratio of viral DNA immunoprecipitated to input DNA for each antibody relative to that of the control antibody. The data present the average of two separate qPCR quantifications from three separate experiments. Significance of changes for the pooled sets of samples from three experiments were analyzed by student's t-test, and the p value is indicated by an asterisk (*p<0.007,**p<0.03).

To confirm that pUL29/28 and/or pUL38 influence the accumulation of IE1 by modulating activity of the MIEP, which controls expression of UL123 mRNA, and to test whether the promoter was controlled by the viral proteins outside the context of the viral genome, we performed assays using a reporter in which luciferase expression is controlled by the MIEP. We have previously demonstrated that pUL29/28 alone can activate the MIEP in HEK-293T cells [41]. Increasing amounts of empty vector or a vector expressing HA epitope-tagged pUL29/28 were transfected together with the reporter into U-2 OS cells, and luciferase activity was determined 48 h later. Increasing amounts of pUL29/28 expression resulted in an ∼7-fold increase in luciferase activity relative to empty vector (Fig. 8A, left upper panel). Using the same lysates that were used to measure luciferase activity, we observed by Western blot analysis the expected increase in the levels of pUL29/28 with increasing input levels of the expression vector relative to cellular tubulin (Fig. 8A, left lower panel). To monitor the possible influence of pUL38 on MIEP activity, we repeated the experiment using U2OS-38 cells expressing pUL38 (Fig. 8A, right lower panel). Transfection of the MIEP luciferase reporter into pUL38-expressing cells resulted in similar levels of luciferase activity as observed in the control cells (Fig. 8A, left upper panel), arguing that pUL38 does not activate the MIEP. In a control experiment, increasing amounts of pUL29/28 increased MIEP activation in the U2OS-38 cells, but not to a greater extent than in cells lacking pUL38. Previously, we demonstrated that expression of pUL38 promotes active mTORC1 kinase and presumably protein translation [36]. To confirm that the increase in luciferase activity is the result of changes in RNA accumulation of the luciferase gene and not translation, we isolated RNA from pUL29/28-expressing U2OS or U2OS-38 cells and quantified the levels of UL29 and luciferase RNA by qRT-PCR. The similar ratios of the viral RNA relative to luciferase RNA (Fig. 8A, right upper panel) demonstrated that the luciferase assay reflected changes in RNA accumulation.

ISRE and NF-κB *cis*-acting elements contribute to full MIEP induction during infection [69], [70]. We tested whether pUL29/28 can activate expression of promoters comprised of these elements to determine whether it functions in part through these specific transcription factor binding sites, and more broadly to ask if the viral protein can influence expression from promoters other than the MIEP. Although it activated the MIEP reporter by a factor of 7, pUL29/28 had relatively little effect on reporter constructs containing promoters controlled by the ISRE or NF-κB elements (Fig. 8B). To confirm that pUL29/28-mediated activation of the MIEP reporter required NuRD components, the experiment was repeated in U2OS cells lacking either CHD4 or RBBP4. The shRNA-mediated knockdowns were confirmed by measuring RNA levels (Fig. 8C, left panel), and MIEP reporter activity in the presence of pUL29/28 in both knockdown cells was reduced to a modest extent but significant in only the CHD4 knockdown samples, as compared with normal U-2 OS cells (Fig. 8C, right panel).

To determine whether this activity could result from a direct interaction of pUL29/28 and elements of the NuRD complex at the MIEP, we performed chromatin immunoprecipitation (ChIP) experiments. Using the *in*UL29F virus, we infected primary fibroblasts and immunoprecipitated crosslinked DNA using an antibody to the MTA2 component of NuRD as well as to the FLAG epitope in pUL29/28. Both antibodies immunoprecipitated more MIEP DNA than a control antibody in three independent experiments (Fig. 8D).

We conclude that pUL29/28 works with components of the NuRD complex to activate the MIEP independently of pUL38, and both proteins interact with the MIEP during infection.

## Discussion

Histone acetylation/deacetylation plays a pivotal role in the regulation of HCMV gene expression [20]–[24]. Analysis of proteins captured with pHDAC1gfp by mass spectrometry predicts that HDAC1 associates with a substantial number of cellular and viral proteins during infection (Fig. 1C and Table S1). HDAC1 exists in different complexes, and the capture assay identified putative interacting proteins that are found within the NuRD, coREST, and Sin3 complexes. We performed reciprocal co-immunoprecipitations to confirm the interaction of pUL38 and pUL29/28 with HDAC1 and to verify that the viral proteins interact with the MTA2 subunit of the NuRD complex (Fig. 2A and B). A relatively lower, but detectable, amount of pUL29/28 was coimmunoprecipitated with a component of the Sin3 complex (Fig. 2C), consistent with the view that the viral protein exists in multiple HDAC1-containing complexes within the infected cell, as predicted by the mass spectrometry analysis.

In addition to these HCMV proteins, we detected peptides corresponding to the viral kinase pUL97 in the HDAC1 capture experiment (Table S1). Although we did not validate this predicted interaction, other herpesvirus-coded kinases have been reported to hyperphosphorylate HDAC1, including the herpes simplex type 1 US3 kinase [56] and the varicella zoster virus ORF66p kinase [71]. Consequently, the possible HDAC1-pUL97 interaction could reflect a kinase-substrate interaction. We failed to detect IE1 or IE2 peptides in the HDAC1 capture experiment. These immediate-early proteins regulate HCMV gene expression, in part, by altering histone acetylation [10], [26], and IE2 has been previously reported to interact with HDAC1 [10] in transfected cells. Our failure to observe IE2 may be in part due to the isolation conditions (e.g. lysis buffer) or poor ionization of corresponding peptides during MS analysis, although we have detected IE2 in other MS analyses that were focused on the immediate-early protein (data not shown). Alternatively, the proteins might interact at the start of infection but not at 24 hpi, when we performed the capture assay.

We have previously shown that pUL29/28 and pUL38 are needed for efficient HCMV replication [37], [39], [41]. Our current results demonstrate that these proteins target the NuRD complex; but, rather than antagonize NuRD (Fig. 6), the viral proteins modulate its activity to support HCMV replication. We observed an ∼10-fold reduction in both HCMV RNA and DNA accumulation in cells expressing shRNAs directed against two different components of the NuRD complex, CHD4 and RBBP4 (Fig. 7C and D). A pUL29/28-deficient virus expressed ∼2-fold-reduced levels of immediate-early RNAs, but accumulated ∼10-fold less DNA when compared to wild-type virus [41]. The more severe effect on viral RNA accumulation observed for a NuRD as compared to a pUL29/28 deficiency suggests that NuRD likely plays a more complex role than simply regulating the MIEP in HCMV replication. The relatively small drop in pUL29/28-induced MIEP reporter activity upon disruption of NuRD components supports this view (Fig. 8C).

Our data demonstrate that components of the NuRD complex, with which pUL29/28 interacts, are needed for efficient replication. This is in contrast to the consequences of drug-mediated, global HDAC inhibition during infection, which enhances immediate-early gene expression [20]–[24], overcomes hDaxx-mediated repression on the MIEP [25] and rescues an IE1-deficient virus [26]. We observed similar increases in IE1 RNA expression in both pUL29-deficient and wild-type virus in the presence of TSA (Fig. 6B), suggesting that the mechanism to overcome hDaxx-mediated repression on the MIEP is still in place in the absence of pUL29/28.

pUL38 interacts with NuRD components only in the presence of pUL29/28 (Fig. 4); and pUL29/28, but not pUL38, can activate the MIEP in a reporter assay (Fig. 8A and B). The NuRD subunit-dependent (Fig. 8C) ability of pUL29/28 to modulate the MIEP in the absence of additional viral proteins argues that it plays a significant role in modulation of the NuRD complex during infection. Further, pUL29/28 failed to significantly activate reporters controlled by ISRE or NF-κB elements (Fig. 8B), supporting the interpretation that that pUL29/28 modulates NuRD activity to generate promoter-specific outcomes.

How does pUL29/28 modulate the NuRD complex to favor expression of the MIEP? The HCMV genome undergoes chromatin remodeling and changes in acetylation during the course of infection through unknown mechanisms [8], [9]. The NuRD complex contains both HDAC and nucleosome remodeling activity [12] and participates in both activating and repressing transcription [72]. As discussed above, global inhibition of HDAC activity enhances viral replication, raising the possibility that NuRD HDAC activity is not important for HCMV. Perhaps pUL29/28 modulates the activity of the MIEP and accumulation of immediate-early RNAs by influencing nucleosome positioning through its interaction with NuRD. In this regard, we observed an interaction of the MTA2 component of NuRD and pUL29/28 with the MIEP (Fig. 8D). There is precedent for a role of nucleosome remodeling in HCMV gene expression. The p150 subunit of the chromatin assembly factor 1 (CAF1) is localized to HCMV replication centers during viral infection [8], and it has been shown to influence MIEP activation [73]. CAF1 consists of three subunits, p150, p60 and p48 (RBBP4), and functions in nucleosome assembly [74] and p150 has been observed in remodeling complexes [75]. Alternatively, a complex of pUL29/28 and NuRD may act globally to alter the nuclear environment. The NuRD complex deacetylates both cellular proteins and histones at cellular promoters [12]. Consistent with a role at sites other than the HCMV genome, pUL29/28 and pUL38 are not restricted to viral replication centers during the course of infection (Fig. 3) [37], [41]. The NuRD complex regulates expression of several cellular genes, including *snail* [76], *wnt4* [77], and *brca1* [78]. Interestingly, expression of *Snail* and *wnt4* are rapidly down regulated during HCMV infection [79]. Beyond histones, NuRD also has been demonstrated to directly deacetylate transcription factors, including p53 and HIF-1α [17], [18]. Casavant *et al*. [80] have argued that p53 is necessary for efficient HCMV replication, influencing viral gene expression [81].

Previously, we have demonstrated that HCMV pUL38 also interacts with and antagonizes the TSC complex during infection [36]. This association is clearly distinct from its association with the NuRD complex, because pUL29/28 is not associated with TSC2 (Fig. 2C). Further, a lower molecular weight isoform of pUL38 binds pUL29/28 and MTA2 than interacts with TSC2 (data not shown). The different forms of pUL38 have been attributed to alternative usage of ATG start codons [36]. In addition, the interaction of pUL38-pUL29/28 with NuRD subunits is evident by 6 hpi (Fig. 5) within the nucleus (Fig. 3), while pUL38-TSC complex is detected after 24 hpi and predominantly within the cytoplasm [36]. The consequences of the pUL38 interaction with NuRD subunits remains unclear. However, our studies suggest that the contribution of NuRD to replication is complex, and we speculate that pUL38 is important in regulating NuRD. Both TSC and NuRD complexes are targets of the human papillomavirus (HPV) E6 and E7 proteins [33], [82]. This conservation in cell targets among different viruses argues for their importance, and consequently for a central role of pUL29/28 and pUL38 in viral pathogenesis.

## Supporting Information

Table S1 List of proteins isolated with HDAC1 at 24 h following infection with HCMV. Protein name, gi number, number of peptides detected in the MS spectra, sequence coverage (%), XProteo score (d'), and amino acid sequences of peptides confirmed by MALDI IT CID are shown for the isolated proteins. Only proteins confirmed by the CID fragmentation of at least two peptides were included in the table. M* indicates an oxidation at methionine; # indicates unique peptides in protein isoforms.
(0.06 MB XLS)

Figure S1 Probing for non-specific isolations due to interactions with the GFP tag or resin. Affinity purification of GFP were performed from fibroblasts expressing free GFP, infected with HCMV, and harvested at 24 hpi. Isolated proteins were resolved by 1-D gel electrophoresis on a 4–12% gradient gel, stained with Coomassie Blue, and identified by mass spectrometry.
(0.42 MB TIF)
