# Supplementary figures and images for: Human Cytomegalovirus UL29/28 Protein Interacts with Components of the NuRD Complex Which Promote Accumulation of Immediate-Early RNA
Source: PLoS Pathog. 2010 Jun 24;6(6):e1000965. doi: 10.1371/journal.ppat.1000965 (PMC2891856; doi:10.1371/journal.ppat.1000965)

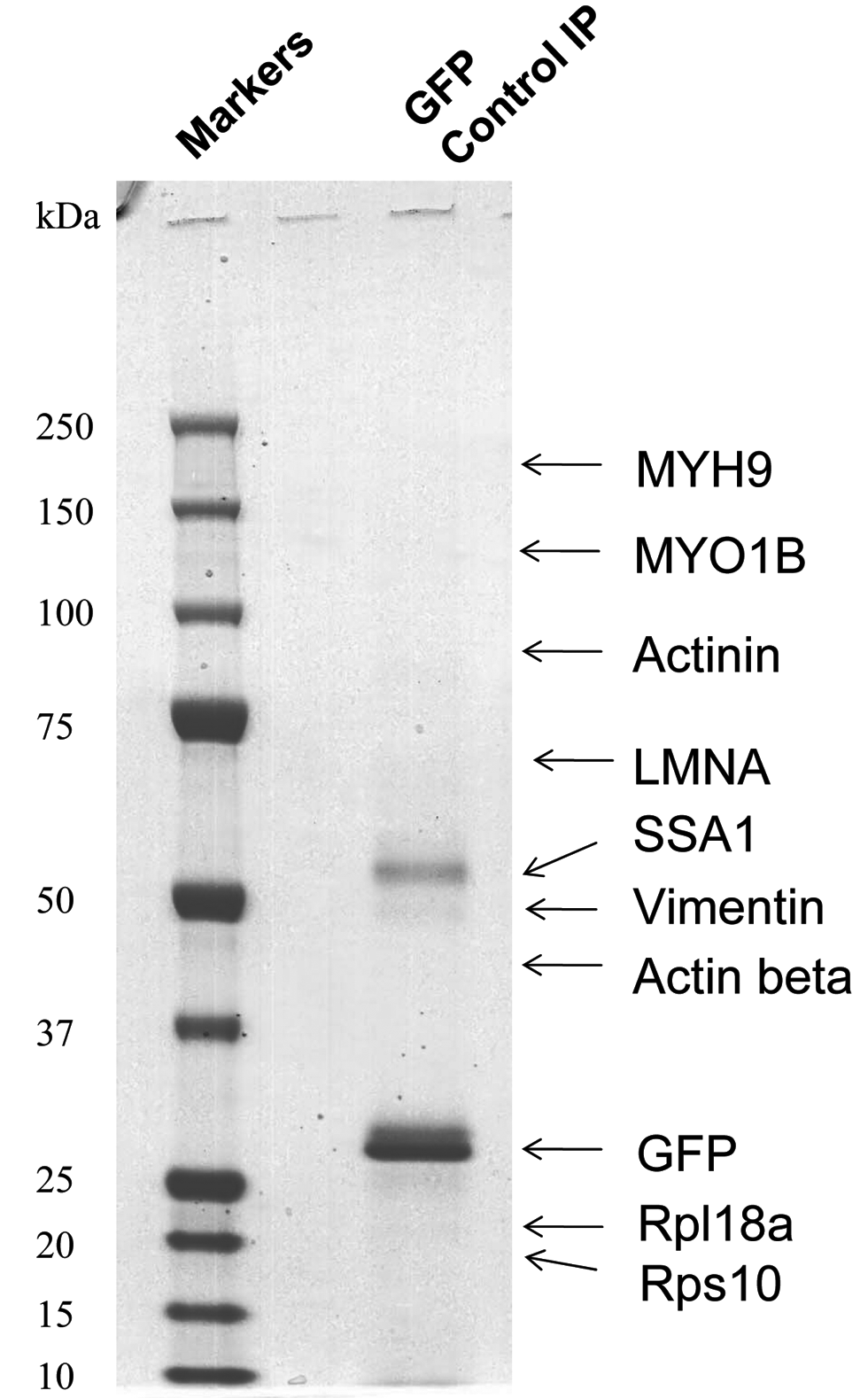

Supplement: Figure S1 — Probing for non-specific isolations due to interactions with the GFP tag or resin. Affinity purification of GFP were performed from fibroblasts expressing free GFP, infected with HCMV, and harvested at 24 hpi. Isolated proteins were resolved by 1-D gel electrophoresis on a 4–12% gradient gel, stained with Coomassie Blue, and identified by mass spectrometry. (0.42 MB TIF) [file ppat.1000965.s002.tif]
